# Natalizumab administration in multiple sclerosis patients during active SARS-CoV-2 infection: a case series

**DOI:** 10.1186/s12883-021-02421-3

**Published:** 2021-11-29

**Authors:** Clara G. Chisari, Simona Toscano, Sebastiano Arena, Chiara Finocchiaro, Arturo Montineri, Francesco Patti

**Affiliations:** 1 Department of Medical and Surgical Sciences and Advanced Technologies “G.F. Ingrassia”, section of Neurosciences, University of Catania, via Santa Sofia 78, 95123 Catania, Italy; 2 Infectious Diseases and Tropical Unit, Azienda Ospedaliero Universitaria “Policlinico-San Marco”, via Santa Sofia 78, 95123 Catania, Italy

**Keywords:** Multiple sclerosis, COVID-19, SARS-CoV-2 active infection, Natalizumab

## Abstract

**Background:**

The Coronavirus disease 2019 (COVID-19) caused by the new Severe Acute Respiratory Syndrome Coronavirus 2 (SARS-CoV-2) has become a pandemic, affecting the therapeutic management for Multiple Sclerosis (MS). Any decision regarding the discontinuation of high-potency agents for moderate and highly active MS should be carefully evaluated, taking into account the potential risk of rebound of the disease. In particular, no data about clinical outcome of patients with MS receiving Natalizumab (NTZ) during active COVID-19 infection have been reported yet.

**Cases presentation:**

We reported on 6 patients treated with NTZ for relapsing MS during active COVID-19 infection, who recovered without reporting any worsening or new symptoms. Most of the patients were asymptomatic, with the exception of one patient who had a slight worst COVID-19 clinical course. No patients received O2-therapy or required intensive care. No neurological complications were observed.

**Conclusions:**

This paper reported the clinical outcome of patients with MS receiving NTZ during active COVID-19 infection. This case series suggests that treatment with NTZ during pandemic is relatively safe and might be continued in selected patients who are infected by COVID-19, thereby reducing the risk of MS disease rebound.

## Background

During the last year, the new Coronavirus Disease 19 (COVID-19) caused by the new Severe Acute Respiratory Syndrome Coronavirus 2 (SARS-CoV-2) has become a pandemic, severely affecting world’s entire population [1]. Older age, male sex, obesity, and presence of comorbidities have been associated to higher risk of mortality due to COVID-19 [2].

People with Multiple Sclerosis (MS) were immediately identified as vulnerable subjects and, more specifically, older age, worse disability, progressive course of MS, comorbidities and male sex were considered as risk factors for more severe COVID-19 course [3–5]. However, although MS patients typically have a higher infection risk due to both the autoimmune pathogenesis of the disease and the frequent use of immunosuppressant drugs, an acceptable level of safety during COVID-19 pandemic has been reported for most of the disease-modifying therapies (DMTs) [6]. Indeed, several studies have suggested that injectables DMTs have a favorable immune profile without an increased exposure risk, while concerns have been raised about the use of oral or intravenous high-potency agents for moderate and highly active MS [6, 7]. Particularly, more recent data have indicated an increased risk of serious infections for patients under B cell–depleting therapies [6].

Despite that, any decision regarding the discontinuation of highly effective DMTs for MS has to carefully take into account the potential risk of disease rebound. For this reason, European Academy of Neurology (EAN), Associazione Italiana Sclerosi Multipla (AISM) and the Association of British Neurologists (ABN) recommended to hold injectable and oral therapies and delay cell-depleting DMTs [8–12]. Moreover, immune-modulating therapies such as the dimethyl-fumarate, teriflunomide, and natalizumab (NTZ) are preferred over cell-depleting drugs during the COVID-19 pandemic for the safer immune profile [11, 12]. Thanks to its non-cell-depleting mechanism of action, it has been supposed that NTZ unlikely increases COVID-19 infection susceptibility and, thus, is likely safe to be administered in patients already treated and also in naïve patients as a new treatment [11, 13]. In addition, data about NTZ infectious side-effect profile have shown a mild increase in the susceptibility to respiratory viral infections [14].

To date, only two cases of COVID-19 in patients with MS treated with NTZ have been reported, both with mild clinical courses and complete recovery [15, 16]. In one of them, the use of the extended interval dosing strategy of 6–8 weeks (already described for lowering the risk of Progressive Multifocal Leukoencephalopathy [PML]) has been proposed for decreasing the infection risk and the access to hospital services during the pandemic [16, 17].

To date, it has been recommended to continue all injectable and oral DMTs and to consider the administration of NTZ in COVID-19 ward for patients infected by COVID-19, in order to avoid a disease rebound [10–12]. Nevertheless, there are still no data about the effects of continuing NTZ treatment during active COVID-19 infection in MS patients.

Here, we reported on six MS patients treated with NTZ during active SARS-CoV-2 infection.

## Cases presentation

Six patients, including 5 females (83.3%), with a mean age of 39.2 ± 9.8 years, were treated with NTZ while they were infected with SARS-CoV-2. Written informed consent from each reported patient was collected.

At the time of the SARS-CoV-2 infection, all patients were negative to John Cunningham virus (JCV)-IgG and the mean number of NTZ doses administered was 41.2 ± 24.3. Four patients received at least one DMT and two patients were treatment naïve before starting NTZ, with only one patient reported clinically relevant comorbidities. Demographical and clinical characteristics of the cohort are summarized in Table 1.

**Table 1:** Demographical and clinical characteristics of patients

Patient	1	2	3	4	5	6
**Age (years)**	47	52	39	31	34	32
**Sex**	female	male	female	female	female	female
**Disease duration (months)**	203	309	165	105	89	167
**Prior therapies**	IFN-beta1b	IFN-beta1a s.c.; IFN-beta1a i.m.; cyclophosphamide	None	None	fingolimod	IFN-beta1a i.m.
**N of relapses at MS diagnosis**	3	2	2	2	3	2
**N of relapses in the year before starting NTZ**	1	2	2	2	1	1
**N of relapses during NTZ**	0	0	0	0	0	0
**EDSS at onset**	4.5	2.5	3.5	1.0	1.5	6.0
**EDSS before at NTZ initiation**	5.0	2.0	3.5	0.0	1.5	4.0
**EDSS before COVID infection**^a^	5.0	4.5	3.5	0.0	0.0	6.0
**N of new/enlarged/Gd-enhanced lesions at MS diagnosis**	0	0	0	2	3	4
**N of new/enlarged/Gd-enhanced lesions at NTZ initiation**	0	0	0	0	0	1
**N of new/enlarged/Gd + −enhanced lesions before COVID-19**^a^	0	0	0	0	0	0
**N of new/enlarged/Gd + −enhanced lesions after COVID-19**^**b**^	0	0	0	0	0	0
**N of NTZ administrations before COVID-19**	28	69	18	13	57	62
**JCV status**^a^	negative	negative	positive (1.07)	negative	negative	negative
**Comorbidities**	none	myxoid liposarcoma ^c^	none	none	none	none
**Smoker**	no	yes	no	yes	no	no
**BMI**	21.6	25.6	24.3	27.1	21.3	25.0

COVID-19: Coronavirus Disease 2019; MS: Multiple Sclerosis; NTZ: natalizumab, IFN: interferon; s.c.: subcutaneously administered; i.m.: intramuscularly administered; EDSS: Expanded Disability Status Scale; Gd: gadolinium; JCV: John Cunningham virus; BMI: body mass index

^a^at last follow-up before COVID-19

b performed after recovery from COVID-19

^c^surgically treated in December 2015

Only two patients reported symptoms suggestive of COVID-19 and underwent nasopharyngeal swab for COVID-19 (Table 2). For the remaining patients, the nasopharyngeal swab was performed for screening purpose before receiving NTZ administration.

**Table 2:** COVID-19 data

Patient	1	2	3	4	5	6
**Date of the first positive oropharyngeal/nasopharyngeal swab**	12/OCT/20	14/NOV/20	13/JAN/21	15/JAN/21	16/JAN/21	05/FEB/21
**Severity of COVID-19**	mild	moderate	mild	mild	mild	mild
**7-point ordinal scale for grading clinical status of COVID-19**	1	3	1	1	2	1
**COVID-19 treatments**	none	paracetamol, azitromicine, prednisolone	none	none	paracetamol	none
**COVID-19 symptoms**	none	fever, sore throat, loss of taste, loss of smell	none	none	headache, bone or joint pain, nasal congestion, loss of taste, loss of smell	none
**Hospitalization**	no	yes	no	no	no	no
**Date of NTZ administration during COVID-19**	26/NOV/20	02/DEC/20	14/JAN/21	20/JAN/21	27/JAN/21	10/FEB/21
**Date of the negative oropharyngeal/nasopharyngeal swab**	30/NOV/20	28/DEC/20	28/JAN/21	16/FEB/21	25/FEB/21	08/MAR/21
**COVID-19 duration (days)**	39	44	15	32	40	31

*NTZ* natalizumab, *COVID-19* Coronavirus Disease 2019

All patients were admitted to the University-Hospital G. Rodolico-San Marco’s COVID-19 ward, using specific bio-containment measures. NTZ (300 mg) was intravenously administered using the extended dosing protocol [18].

Most of the patients were asymptomatic (7-point ordinal scale for grading clinical status = 1) and were admitted to the hospital for the sole purpose of being administered with NTZ, with the exception of one patient (7-point ordinal scale for grading clinical status = 3), who was hospitalized because of his worsening of the respiratory symptoms. This patient had been previously treated with three DMTs before starting NTZ and reported a previous comorbidity, a mixoid liposarcoma in the right leg, surgically removed in December 2015. The patient did not undergo any chemotherapy.

No patients received O2-therapy or required intensive care. Five of six patients were discharged to home-quarantine after 3-4 days from the hospitalization and recovered from COVID-19 after about a month (33.5 ± 10.3 days), with no abnormalities in blood tests. For just one patient, the clinical course was slightly longer (44 days) compared to other patients.

After the hospital discharge, all patients underwent a neurological follow-up visit showing clinically stable condition, with neither relapses nor EDSS worsening. Moreover, no enlarging T2 lesions or new gadolinium-enhanced lesions were observed at the Magnetic Resonance Imaging (MRI) examination performed after recovery from COVID-19.

No respiratory or neurological symptoms have been reported since the hospital discharge. PCR on nasopharyngeal swab was double-negative in all patients. The detection of antibodies against SARS-Cov-2 has not been performed yet.

NTZ was re-administered six weeks later, according to the extended interval dosing, without any complication.

## Discussion and conclusions

This paper reported the clinical outcome of patients with MS receiving NTZ while they were infected by SARS-CoV-2. Currently, there are no data guiding health professionals to manage MS treatments during active SARS-CoV-2 infection. Despite the lack of clinical studies, the EAN, AISM, and ABN recommend to continue all injectable and oral DMTs in patients positive for SARS-CoV-2; especially interferons, whose potential antiviral should be also considered [10–12]. According to these recommendations, the concern about a rebound disease activity secondary to NTZ-discontinuation should be kept in mind, individualizing the treatment strategy, such as extended-interval dosing, based on the clinical situation. A recent online survey, promoted by the European Committee for Treatment and Research in Multiple Sclerosis (ECTRIMS), has explored the impact of COVID-19 emergency on MS treatment practices, showing that 70% of the neurologists reported changes in DMT management. In particular 18-43% of them would postpone the retreatment with alemtuzumab, cladribine and anti-CD20, while the 64% would not modify NTZ-infusion schedule [19].

Since the COVID-19 has become pandemic, it is common practice in our MS center for all patients to undergo a COVID-19 nasopharyngeal swab test no more than 72 h prior to the medical service (i.e. follow-up visit, MRI, administration of NTZ dose or other intravenous DMT, etc.). This, together with the possibility to admit patients with confirmed SARS-CoV-2 infection to a COVID-19 ward for their NTZ treatment, allowed to isolate COVID-19 cases before coming into contact with other patients.

In our case series, only one patient had a more severe clinical course; however, the contribution of NTZ therapy to his outcome cannot be demonstrated as he had multiple risk factors for severe COVID-19. Particularly, several studies demonstrated that older age is an independent risk factor of severe COVID-19 [5, 6, 19, 20]. In addition, male sex, habit of smoking, and comorbidities (myxoid sarcoma previously surgically treated) may have contributed to the worse clinical course of COVID-19 in this patient. Nonetheless, this patient fully recovered from all COVID-19 symptoms.

In the current literature, both cases of SARS-CoV-2 infection in patients with MS treated with NTZ reported a benign clinical course with complete recovery. Similar to our patients, no worsening of MS clinical conditions was observed, even after the recovery from COVID-19 [15, 16]. Particularly, the Authors have suggested that the low viral replication rate, the repeated negative results with PCR for SARS-CoV-2, as well as the benign course of the disease observed in these patients, may be explained by the NTZ mechanism of blockade of integrins, as SARS-CoV-2 may enter human cells through these receptors. According to this theory, the Authors also concluded that the use of drugs that blocking integrins may represent a therapeutic option against SARS-CoV-2 infection [15].

Interestingly, recent studies reported a decrease in the total number of natural killer (NK) cells and CD8^+^T lymphocytes in patients with COVID-19 [13, 21]. Particularly, the NK cell count reduction was more marked in patients with more severe COVID-19 disease compared to milder cases. Since NTZ induce an increase in circulating NK cells by reducing their migration into the CNS, it also may potentially represent an alternative treatment for COVID-19 [22, 23].

It should be also noted that one fatal case of COVID-19 was described in a 51-year-old African American woman with MS on NTZ treatment. However, because of the presence of multiple risk factors for severe COVID-19 (race, obesity, hypertension, and elevated inflammatory markers), it is unclear whether NTZ may have played a role on it [24]. In addition, several concerns have been raised about the effect of NTZ in reducing the lymphocytes migration to the lungs and mucosa that may potentially increase the viral shedding [25]. NTZ also reduces immune surveillance in the CNS and, thus, as shown for PML, may potentially increase the risk of COVID-19 encephalitis [25].

Notwithstanding these concerns, a recent Italian multicenter study of the Musc-19 group confirmed that NTZ has shown an acceptable safety profile during the COVID-19 pandemic, with no significant increased risk of severe COVID-19 course, even after adjusting for several confounders [6].

Another study recently investigated self-reported COVID-19 symptoms and disease severity in MS patients under NTZ or fingolimod with serology confirmation for SARS-CoV-2 infection. In this study 13.4% patients on NTZ or fingolimod had anti-SARS-CoV-2 antibodies, showing no or only mild COVID-19 symptoms and, thus, supporting the hypothesis that it is safe to continue treatment with these drugs in the current setting [26].

Furthermore, in the light of the recent development of new SARS-CoV-2 viral protein and inactivated vaccines, it is also conceivable that NTZ may induce an adequate response to the COVID-19 immunization, as suggested from several influenza vaccine studies [27, 28].

In conclusion, MS is a chronic and severe disease with high risk of disability accumulation especially in patients with more aggressive course, who require more aggressive treatment approach from the beginning of the therapeutic management [29]. Particularly, NTZ has proved highly effective, both in clinical trials and in real-word setting, reducing the rate of clinical relapses and slowing the disease progression [30]. However, during the COVID-19 pandemic, it was observed that NTZ administrations have been discontinued or delayed because of fear of more severe course of COVID-19 [19].

Our cases series may encourage Clinicians to not discontinue or delay NTZ administration and/or to not switch to different DMT in selected patients with active SARS-CoV-2 infection. Thus, it could be suggested to continue NTZ therapy per protocol in COVID-19 wards, using specific bio-containment measures.

Despite the fears associated with the admission to the COVID-19 ward, in our cases study all patients accepted to be hospitalized and were dismissed after about 3-4 days.

In conclusion, although the limit of the small sample size, our single-center experience shows that treatment with NTZ during pandemic is relatively safe and could be continued in selected patients who are infected by SARS-CoV-2, thereby reducing the risk of MS rebound commonly reported after NTZ discontinuation.

## Data Availability

Dataset is available under reasonable request.

## Abbreviations

ABN: Association of British Neurologists
AISM: Associazione Italiana Sclerosi Multipla
COVID 19: Coronavirus Disease 19
DMT: Disease-modifying therapy
EAN: European Academy of Neurology
JCV: John Cunningham virus
MS: Multiple Sclerosis
NK: Natural killer
NTZ: Natalizumab
PML: Progressive Multifocal Leukoencephalopathy
SARS-CoV-2: Severe Acute Respiratory Syndrome Coronavirus 2
